# Study on the mechanism of plant metabolites to intervene oxidative stress in diabetic retinopathy

**DOI:** 10.3389/fphar.2025.1517964

**Published:** 2025-02-05

**Authors:** Tianyao Gong, Dongmei Wang, Jinyan Wang, Qun Huang, Haiyan Zhang, Chunmeng Liu, Xinglin Liu, Hejiang Ye

**Affiliations:** ^1^ Eye School of Chengdu University of Traditional Chinese Medicine, Chengdu, China; ^2^ School of Basic Medical Sciences, Chengdu University of Traditional Chinese Medicine, Chengdu, China; ^3^ Hospital of Chengdu University of Traditional Chinese Medicine, Chengdu, China; ^4^ School of Management, Chengdu University of Traditional Chinese Medicine, Chengdu, China

**Keywords:** plant metabolites, oxidative stress, diabetic retinopathy, polyphenols, polysaccharide, saponins, alkaloids

## Abstract

Diabetic retinopathy is the main microvascular complication of diabetes and the first blinding eye disease in the working-age population. Oxidative stress is an important pathogenesis of diabetic retinopathy. Plant metabolites can be divided into two types: primary metabolites and secondary metabolites, secondary metabolites are the main active components and important sources for developing new drugs. It has unique effect in the treatment of diabetic retinopathy. However, the research on the intervention mechanism of plant metabolites in diabetic retinopathy are still relatively shallow, which limit the application of plant metabolites. With the deepening of research, more and more plant metabolites have been reported to play a role in treating diabetic retinopathy through anti-oxidative stress, including polyphenols, polysaccharides, saponins, alkaloids, etc. Therefore, this article reviewed the potential of plant metabolites in the treatment of diabetic retinopathy in the last 10 years and elucidated their mechanism of action. We hope to provide some references for the application of plant metabolites and provide valuable resources for the research and development of new drugs for diabetic retinopathy.

## 1 Introduction

Diabetes mellitus is a chronic disease characterized by hyperglycemia caused by insulin deficiency or destruction of islet beta cells (Khatik et al., 2018). The number of people with diabetes worldwide is expected to increase by 46%–783 million by 2045 (International Diabetes Federation, 2021). Diabetic Retinopathy (DR), one of the major complications of diabetes, affects the retinal microvascular system and is the leading cause of blindness in people of working age. DR Is divided into non-proliferative diabetic retinopathy and proliferative diabetic retinopathy. Non-proliferative diabetic retinopathy may further evolve into proliferative diabetic retinopathy, which can lead to various vision-threatening complications such as vitreous hemorrhage, intravitreal neovasculogenesis, retinal traction detachment, and even blindness (Flaxel et al., 2020). In the coming decades, the global incidence and disease burden of DR Will increase substantially, from an estimated 103 million people in 2020 to 161 million people in 2045 (Teo et al., 2021; Kang and Yang, 2020). This will make DR One of the major global public health burdens.

DR is a multifactorial disease with a complex pathogenesis, including inflammation (Wu et al., 2020), oxidative stress (Ortega, 2021), VEGF (Wu et al., 2020), etc. Current treatments focus on advanced DR, when blood vessels have been severely altered and retinal neurons have been irreparably damaged (Rodríguez et al., 2019). Treatment methods include vitreoretinal surgery, laser photocoagulation, intravitreal injection of anti-VEGF drugs, and standard biomedical treatment (Sivaprasad et al., 2017). Among these therapies, retinal photocoagulation surgery and intravitreal anti-VEGF therapy are still the main treatments for proliferative diabetic retinopathy (Flaxel et al., 2020). Although anti-VEGF therapy and laser photocoagulation therapy can “improve” vasculopathy and DR Severity, reports show that underlying retinal ischemia does not change, and lesions and retinopathy usually recur quickly after cessation (Couturier et al., 2019; Pearce et al., 2020). The cost of surgery is expensive, the prognosis is uncontrollable, and the effect of standard biomedical treatment alone is not ideal. Therefore, the development of safer and more economical hypoglycemic drugs has become an urgent problem that researchers need to solve.

As the advantages of botanical drugs in the prevention and treatment of chronic diseases continue to emerge, a variety of botanical drugs with mild side effects are used to treat DR (Li et al., 2023; Xu et al., 2018; Song et al., 2019). A review by Ai et al. (2020) showed that botanical drugs could significantly improve DR Plant metabolites generally refer to all metabolites produced in the plant body, including primary and secondary metabolites. Primary metabolites are essential for plant growth and development, such as carbohydrates, fats, proteins, etc. Secondary metabolites are non-essential small molecule compounds produced in the normal growth process of plants, with antioxidant, anti-inflammatory, antibacterial, antiviral and other biological activities, such as alkaloids, terpenes and so on. Natural products refer to various substances extracted from nature, including the components of plants, animals, microorganisms and other organisms or their metabolites. Thus, plant metabolites are part of natural products, but natural products also include substances of non-plant origin.

The results showed that plant metabolites had good effect and little side effect in treating DR (Wang L. et al., 2023; Zhang et al., 2019a; Niu et al., 2023). It has inhibitory effects on a variety of diabetes targets, including α-glucosidase, α-amylase, and peroxisome proliferator-activated receptor γ (Ferrannini et al., 2005; Matsui et al., 2001). Polyphenols are bioactive molecules that can be extracted from common plants and have become the subject of research interest in recent years due to their significance in the treatment of diseases such as diabetes and human health-related diseases (Pallavi et al., 2018). It has the function of anti-oxidation, strengthening blood vessel walls, reducing blood fat, increasing body resistance, preventing arteriosclerosis, and so on Joma et al. (2024), Shen et al. (2022). Resveratrol (Huang et al., 2020) and multiple flavonoids are all polyphenolic metabolites. Multiple polyphenols have been shown to inhibit alpha-glucosidase and alpha-amylase with inhibition levels of 50% or higher (El-Beshbishy and Bahashwan, 2012). Natural polysaccharides exist widely in nature, including plants, Marine organisms, and microorganisms. Polysaccharide is a kind of carbohydrate substance with a complex molecular structure, widely existing in animal cell membranes, and plant and microbial cell walls, and is one of the four basic substances constituting life. It mainly has the functions of immune regulation, anti-tumor, anti-oxidation, anti-virus, lowering blood sugar, lowering blood lipids, and delaying aging (Benalaya et al., 2024; Wu et al., 2016). Lycium barbarum polysaccharide, Astragalus polysaccharide, Angelica polysaccharide, and Notoginseng polysaccharide are all polysaccharides, which have antioxidant, immunomodulatory, and anti-tumor effects. Saponins are secondary plant metabolites, which contain triterpenoid aglycones or steroidal aglycones with uronic acid. It has a variety of biological activities, including anti-inflammatory, antioxidant, anti-glucose, and immunomodulatory functions (Shen et al., 2023), including ginsenosides, Panax notoginseng saponins, and so on. Alkaloid is a kind of nitrogen-containing alkaline organic metabolites that exists in nature and is one of the most important metabolites of botanical drug. It has a variety of pharmacological activities, including hypolipidemic, anti-inflammatory, anti-infection, and anti-tumor effects (Yu et al., 2022), including evodia, berberine, marine, and so on. Reactive Oxygen Species (ROS) has been recognized as a potential causative factor in diabetic retinopathy. Research shows that (Fanaro et al., 2023; Wang C. et al., 2022; Gupta et al., 2011) plant metabolites may ameliorate the symptoms of diabetic retinopathy mainly by regulating ROS. In this study, we conducted a literature search using databases such as CNKI (https://www.cnki.net/), Wanfang (https://www.wanfangdata.com.cn/), PubMed (https://pubmed.ncbi.nlm.nih.gov/), and Web of Science (https://www.webofscience.com/), using search terms like “plant metabolites” OR “Chinese herbal metabolites” AND “diabetic retinopathy” to identify articles related to “oxidative stress” published in the past decade. We aimed to summarize plant metabolites that act on diabetic retinopathy through the oxidative stress (OS) pathway, hoping to provide data references for the development of new therapeutic drugs for diabetic retinopathy.

## 2 Relationship between oxidative stress and diabetic retino-pathy

### 2.1 Oxidative stress and diabetic

Oxidative stress plays a crucial role in the occurrence and development of diabetes. Particularly under hyperglycemic conditions, the level of oxidative stress significantly increases. Oxidative stress refers to the situation where free radicals in the body, especially ROS, are produced in excess and exceed the clearance capacity of the antioxidant defense system, leading to cell and tissue damage. Diabetic patients often have hyperglycemia, and long-term hyperglycemia is considered one of the main factors exacerbating oxidative stress. When excessive glucose undergoes autoxidation in the body, free radicals can be generated. These free radicals not only directly damage islet β cells and reduce insulin secretion but also promote the exacerbation of oxidative stress through the polyol pathway and glycosylation reaction (Darenskaya et al., 2021).

The influence of oxidative stress on diabetes is primarily manifested in insulin resistance and the occurrence of complications. To begin with, oxidative stress undermines the function of pancreatic islet β cells, which leads to a reduction in insulin secretion and further exacerbates the elevation of blood glucose levels. Moreover, oxidative stress disrupts the action of insulin by modulating cell signaling pathways, causing the sensitivity of tissue cells to insulin to decline and thus giving rise to insulin resistance. Additionally, oxidative stress is closely associated with microvascular disorders and cardiovascular complications in diabetes. It facilitates inflammatory responses and arteriosclerosis through damaging the vascular endothelium (Papachristoforou et al., 2020). Consequently, oxidative stress serves as not only one of the pathological foundations of diabetes but also an accelerating element in the development of diabetic complications.

### 2.2 Oxidative stress and diabetic retinopathy

Oxidative stress is an imbalance of the body caused by the incongruity of the generation and removal of free radicals. Overproduction of ROS can affect the activity of endogenous antioxidant enzymes, such as superoxide dismutase (SOD), glutathione peroxidase (GPx), catalase (CAT), etc., which leads to the decreased ability to protect cells. It causes the imbalance of the oxidation-antioxidant system and promotes the occurrence and development of DR. The outer layer of the human retina is rich in a variety of unsaturated fatty acids, which play an important role in the structure and energy of the retina (Tang and Tan, 2023). However, the imbalance of oxidation-antioxidant regulation in the body and the excess of ROS will lead to the oxidation and degradation of these fatty acids, and at the same time start the lipid peroxidation reaction, which will affect the physiology of the retina and the normal function of visual cells. The complex physiological functions of the retina require long-term high oxygen consumption to support, so it is easier to produce ROS, accelerate the process of lipid peroxidation, and ultimately accelerate the progress of DR. At the same time, excessive ROS accumulation can also damage the tissues in and around the retinal blood vessels so that the repeated interaction eventually leads to the occurrence of DR.

The occurrence and development of diabetic retinopathy are closely related to the injury, apoptosis, abnormal proliferation of vascular endothelial cells, the breakdown of cell connections, and the proliferation of new blood vessels after ischemia. There is a complex relationship between oxidative stress and various pathogenesis of diabetic retinopathy, such as inflammation, mitochondrial dysfunction, and apoptosis (Tian and Li, 2024). The retinal oxidative damage induced by hyperglycemia mainly involves four metabolic pathways: advanced glycation end products (AGE) pathway, polyol pathway, hexosamine pathway, and protein kinase C pathway (Shi, 2021). In addition, the phenomenon of “metabolic memory” caused by irregular epigenetic modification in diabetic patients has also been shown to be related to the overproduction of ROS (Sui et al., 2020). The metabolism of high sugar generates a large number of reactive oxygen species, which damages the retina, leads to oxidative damage of DNA, and eventually leads to apoptosis. At the same time, the increase in the level of oxidative stress activates cytokines and further upregulates the level of oxidative stress, thus causing the structural and functional abnormalities of retinal cells.

### 2.3 Mitochondrial dysfunction caused by oxidative stress in diabetes mellitus

Mitochondria play an important role in cells by metabolizing nutrients and producing adenosine triphosphate (Kim et al., 2008). Mitochondria are also important sources of ROS in most mammalian cells (Murphy, 2009). In diabetic conditions, excess ROS can damage mitochondrial proteins/enzymes, membranes, and DNA, resulting in disruption of mitochondrial adenosine triphosphate production and other essential functions. Mitochondrial dysfunction caused by oxidative stress in diabetes can be divided into the following aspects: 1. In the condition of diabetes, excess ROS will weaken the antioxidant defense ability of cells, damage mitochondrial DNA, and proteins, and thus affect mitochondrial function (Murphy, 2009; Dröge, 2002). 2. The increase of oxidative stress will damage the inner membrane of mitochondria, resulting in decreased membrane potential, which will affect the mitochondrial respiratory chain and other mitochondrial metabolites, leading to further decline in mitochondrial function, and ultimately leading to mitochondrial fragmentation, further decline in oxidative phosphorylation and increase in ROS production (Kowaltowski and snd Vercesi, 1999; Maassen et al., 2004). 3. ROS can disrupt cell signaling and cause mitochondrial dysfunction, resulting in insufficient energy and eventually loss of function (Juan et al., 2021).

### 2.4 Oxidative stress-related factors and diabetic retinopath-y

#### 2.4.1 Inflammatory factors

Some studies believe that DR Is a low-grade chronic inflammatory disease, the release of pro-inflammatory cytokines and the adhesion of white blood cells to retinal capillaries are the initial events in the development of DR (Yu and Li, 2018). IL-6 and TNF-α are the core factors in the inflammatory cascade. IL-6 aggravates the condition of diabetic patients by damaging the body’s islet beta cells and affecting the normal secretion of insulin. TNF-α and CRP stimulate retinal vascular endothelial cells to produce oxygen free radicals, increase the release of lipid peroxidation products, and cause retinal microangiopathy. The study found that the levels of inflammatory cytokines (including IL-1, IL-6, IL-8, IL-17, and TNF-α) in aqueous humor were increased in patients with DR and may be synergistically involved in the pathogenesis of DR (Feng et al., 2018). The increase of TNF-α, CRP, and IL-6 was the most obvious in the proliferative stage of DR Patients, followed by the non-proliferative stage retinopathy patients. IL-1β is also an interleukin factor closely related to DR. IL-1β and TNF-α jointly inhibit the expression of retinoid-binding protein between photoreceptors, and causing degeneration of retinal neurons and capillary blood vessels, thus increasing the degeneration of neurons and capillaries of DR (Jin et al., 2023). The NLRP3 inflammasome is a multi-protein complex that exists inside cells, It consists of NLRP3 receptor protein, Apoptosis-associated speck-like protein containing a CARD (ASC), and the effector procysteine aspartate-specific protease 1(Pro-Caspase-1) consists of three parts. The NLRP3 inflammasome is an important part of the innate immune system, which promotes the activation of aspartate proteolytic enzyme 1(Caspase-1) and the secretion of the pro-inflammatory cytokine IL-1βor IL-18 (Feng et al., 2018).

#### 2.4.2 Oxidative stress index

MDA (malondialdehyde), SOD (superoxide dismutase), ROS and HO-1 (heme oxygenase-1) are the most commonly used indicators of free radical levels. MDA is the product of lipid peroxidation. In diabetic retinopathy, MDA is increased correspondingly, and the increase of MDA level can reflect the degree of oxidative damage of retinal tissue. SOD is an important part of the antioxidant defense system of retina. In the early stage of diabetic retinopathy, due to the increased production of ROS, the body may fight oxidative stress by up-regulating the expression and activity of SOD. In the condition of long-term hyperglycemia, the activity of SOD will be reduced due to glycosylation modification and other factors, which will aggravate the damage of the retina caused by oxidative stress. In the condition of diabetes, hyperglycemia is the main cause of increased ROS production, and excessive ROS will cause damage to various retinal cells such as retinal pigment epithelial cells, ganglion cells, and endothelial cells. HO-1 plays an important cytoprotective role in diabetic retinopathy. When stimulated by oxidative stress and inflammation, HO-1 expression in retinal cells increases. HO-1 catalyzes the decomposition of heme to produce products such as biliverdin, carbon monoxide (CO) and free iron, in which biliverdin is converted to bilirubin and is a potent antioxidant that can clear ROS. Through its metabolites, HO-1 can also inhibit the formation of retinal neovascularization and reduce the degree of lesions (Yu and Li, 2018).

In conclusion, in diabetic retinopathy, ROS is an important factor leading to the lesion, SOD and HO-1 are important defense mechanisms against oxidative stress, and MDA is an important marker reflecting the degree of oxidative damage of retinal tissue. These indicators are of great significance for the study of the pathogenesis, diagnosis and treatment of diabetic retinopathy (Yang and Song, 2023).

#### 2.4.3 Vascular endothelial cytokines

##### 2.4.3.1 Vascular endothelial growth factor (VEGF)

VEGF is a key regulator of angiogenesis, has a highly specific endothelial cell mitogen, and is closely related to DR Neovascularization (Gao et al., 2017). The family includes VEGF-A, VEGF-B, VEGF-C, VEGF-D, VEGF-E, VEGF, and PLGF (placental growth factor). When VEGF is highly expressed, the tight connection between endothelial cells in the inner layer of the retina is destroyed, and the vascular permeability of the retina is increased, resulting in retinal vascular leakage, which leads to the destruction of the blood-retinal barrier and the formation of new vessels. Inhibition of VEGF expression is critical to slow down the progression of DR, so VEGF is one of the key targets in studying the early lesions and retinal protection of DR (Jin et al., 2023).

##### 2.4.3.2 Hypoxia inducible factor-1α (HIF-1α)

HIF-1α is a part of the transcription complex, which can promote oxygen transport, angiogenesis, metabolic adaptation, cell proliferation, and survival stress. HIF-1 expressed under hypoxia can promote blood vessel formation by increasing the expression of a large amount of VEGF, thus affecting the transport of oxygen to the inner cells (Chai et al., 2020).

##### 2.4.3.3 Serum hepatin-A1 (Ephrin-A1), C1q tumor necrosis factor-associated protein 9 (CTRP9)

Ephrin-A1 is a hepatocyte family protein that promotes angiogenesis and promotes retinal neovascularization through mediating the expression of vascular growth factor. CTRP9 is an adipokine with high homology to adiponectin, which has the function of protecting vascular endothelial cells and is closely related to body obesity. Abnormal expression of serum Ephrin-A1 and CTRP9 in diabetic retinopathy patients. In addition, serum Ephrin-A1 in proliferative diabetic retinopathy patients was higher than that in non-proliferative diabetic retinopathy patients, and CTRP9 level was lower than that in non-proliferative diabetic retinopathy patients (Gao et al., 2017; Zhou et al., 2021).

##### 2.4.3.4 Correlation between inflammatory factors, oxidative stress, and endothelial factors

**FIGURE 1:**
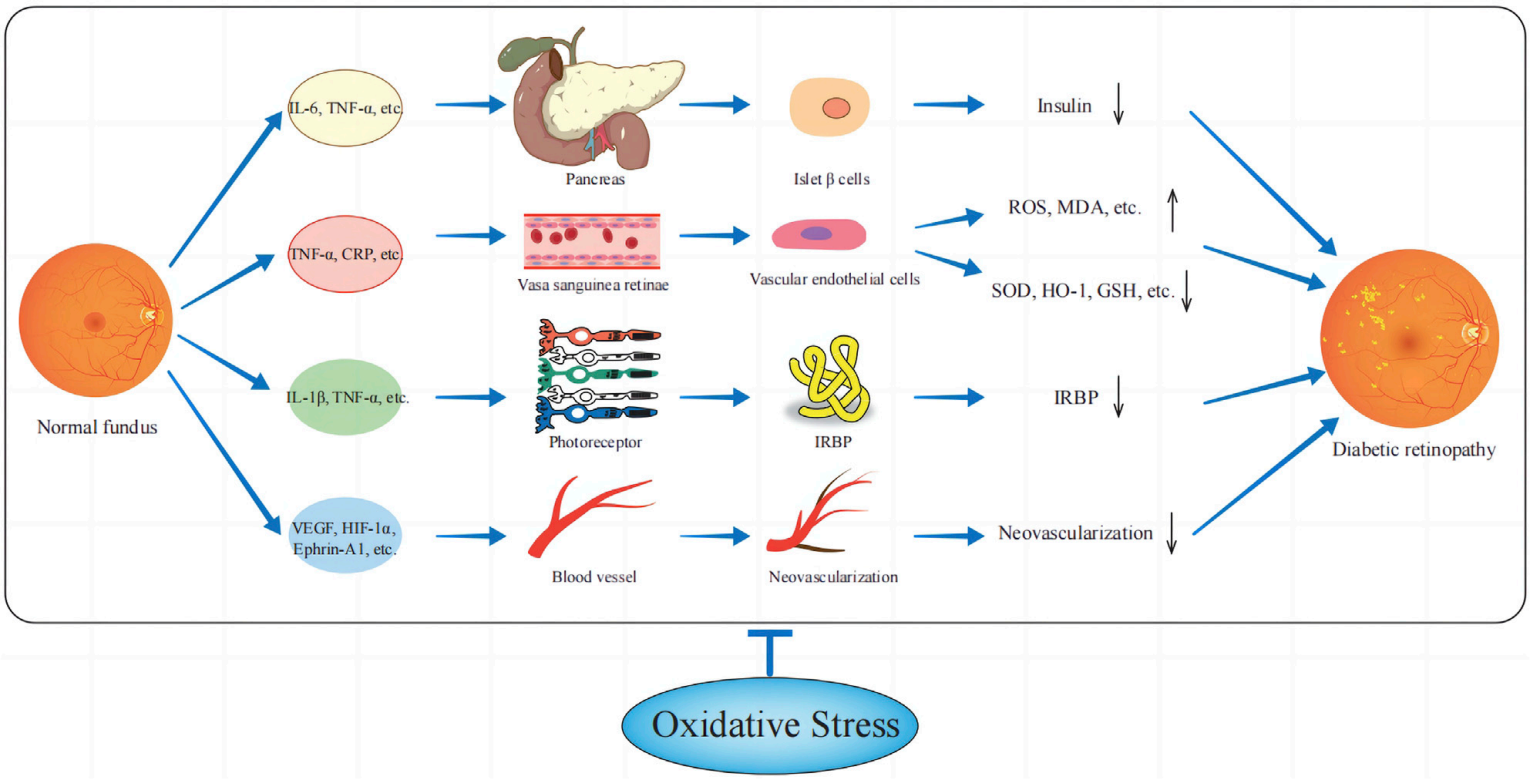
Relationship between oxidative stress-related factors and diabetic retinopathy. Inflammatory factors and vascular endothelial factors act on pancreatic βcells, retinal vascular endothelial cells, photoreceptor cells, etc., increase ROS, and MDA, reduce GSH, SOD, etc., and make the patient’s body in a state of oxygen-free radical overload, thereby promoting diabetic retinopathy.

Overall, a complex interaction network is formed between inflammatory factors, oxidative stress, and endothelial factors. During the onset and progression of DR, the release of inflammatory factors triggers oxidative stress, which in turn promotes the increase of inflammatory factors and the expression of VEGF (Brzović-Šarić et al., 2015; Andrés-Blasco et al., 2023). This process exacerbates retinal neovascularization and increases vascular permeability. Ultimately, these factors work together to drive microvascular changes in the retina, the breakdown of the blood-retinal barrier, and neurodegenerative changes in the retina, thereby worsening the pathological damage of DR (Figure 1).

## 3 Application of plant metabolites in reducing oxidative stress in the treatment of diabetic retinopathy

Plant metabolites are all metabolites produced during plant growth and development, which has a clear chemical structure and a variety of pharmacological activities, which can intervene in diseases through multiple targets, and is an important source of new drug research and development. Plant metabolites have the advantages of precise efficacy, multi-target intervention, and low toxicity and side effects. At the same time, Plant metabolites have the advantage of a clear structure of chemical drugs, which can be replicated and promoted, providing important ideas for the research and development of new drugs (Qiu et al., 2021).

Diabetic retinopathy in traditional Chinese medicine belongs to the category of “thirst-quenching cataract” and “thirst-quenching eye disease”, and the pathogenesis is mainly liver and kidney deficiency, qi and yin deficiency, which can be combined with phlegm turbidity and blood stasis (An et al., 2024). More and more studies have shown that plant metabolites can act on diabetic retinopathy through multiple targets, and the mechanism of oxidative stress may be the main mechanism (Tian and Li, 2024). Therefore, we try to summarise the plant metabolites that can act on DR through the OS pathway, hoping to provide data reference for the development of new drugs for DR. To facilitate the study of the structure and mechanism of action of plant metabolites, we divided the plant metabolites with potential for the treatment of DR into four categories according to their different chemical structures: polyphenols, polysaccharides, saponins, and alkaloids.

### 3.1 Polyphenols

Plant metabolites of polyphenols can be divided into flavonoids and non-flavonoids. Flavonoids are based on 2-phenylchromone as the basic parent nucleus, and its basic structure is A C6-C3-C6 skeleton composed of two benzene rings (A ring and B ring) interconnected through the central three-carbon chain, such as quercetin and puerarin, which mainly have antioxidant, anti-inflammatory and cardiovascular protection effects. Non-flavonoids are mainly stilbenes and phenolic acids, such as picotaxol and ferulic acid, which mainly have antioxidant, anti-platelet aggregation and anti-cancer effects (Table 1).

**TABLE 1:** Application of polyphenols in reducing oxidative stress in the treatment of diabetic retinopathy.

Polyphenols	Chemical formula	Models	Administration	Mechanism	Reference
Curcumin	C21H20O6	RPE cells	10 μmol/L	↓: TNF-α, IL-6, IL-1β, ROS, Akt, mTOR phosphorylation	Zhang and Wang (2019)
		STZ-induced diabetic rats	200 mg/kg/day	↓: MDA, HO-1, Nrf2; ↑: SOD, T-GSH	Ran et al. (2019)
Puerarin	C21H20O9	STZ-induced diabetic rats	100 mg/kg	↓: MDA, IL-1β, TNF-α, MDA, Nrf2, p-ERK, P53; ↑: SOD, Bcl-2	Chen et al. (2022)
Wogonin	C16H12O5	STZ-induced diabetic rats	200 mg/kg	↓: VEGF, CTGF; ↑: SOD	Yang et al. (2021)
Luteolin	C15H10O6	STZ-induced diabetic rats	50 mg/kg	↓: MDA, SOD, NLRP1, NOX4, TXNIP, NLRP3	Cheng et al. (2019)
Quercetin	C15H10O7	STZ-induced diabetic rats	100 mg/kg	↓: MDA, 8-OHdG, TNF-α, IL-6, IL-β, Bax, p-p38MAPK; ↑:SOD, Bcl-2	Wang et al. (2021)
		STZ-induced diabetic rats	10、50 mg/kg	↓: MDA, Bax, P53; ↑: SOD	Kumar et al. (2014)
		STZ-induced diabetic rats	25、50 mg/kg	↓: TNF-α, IL-1β, NF-kB, caspase-3, GFAP, AQP4; ↑: GSH, SOD, CAT	Song et al. (2016)
Blueberry anthocyanins		STZ-induced diabetic rats	20、40、 80 mg/kg	↓: ROS, MDA, TNF-α, IL-1β; ↑: GSH, GPx,HO-1, Nrf2	Jiménez-Osorio et al. (2015)
Resveratrol	C14H12O3	Human Retinal Endothelial Cells	1 μM	↓: ROS, EndMT, NADPH	Kotake et al. (1998)
		Bovine retinal capillary endothelial cells	1、5、10、20 μM	Activation of the AMPK/Sirt1/PGC-1α pathway; ↓: ROS	Giordo et al. (2021)
Piceatannol	C14H12O4	STZ-induced diabetic rats	200 mg/kg	↓: MDA; ↑: SOD, Nrf2, HO-1	Fan et al. (2017a)
Astragaloside IV	C41H68O14	STZ-induced diabetic rats	80 mg/kg/day	↓: NF-κB; ↑: Nrf2	He et al. (2014)
		STZ-induced diabetic rats	50、100、200 mg/kg	↓: MDA, IL-6, TNF-α, VEGF; ↑: SOD	Li et al. (2024)
		STZ-induced diabetic rats	0.02、0.04、0.08 g/kg	↓: IL-6, TNF-α, IL-1β, VCAM-1, HIF-1α, VEGF-A; ↑: GSH, SOD, CAT	Yu et al. (2020)
		rats RCECs	5、10、20 μM	↓: ROS, MDA, Nox4, NADPH; ↑: SOD, MnSOD, CAT, GSH-PX,GSH	Jiang et al. (2023)
wogonoside	C22H20O11	hRMECs; STZ-induced diabetic rats	30 μmol/L; 10、20、30、40 μmol/L	↓: ROS, NO, IL-1β, IL-6, VEGF, HIF-1α; ↑: GSH-ST, SIRT1	Du et al. (2024)
Paeonol	C9H10O3	STZ-induced diabetic rats	50、100、200 mg/kg	↓: MDA; ↑: GSH, SOD, CAT	Tian et al. (2019)

Curcumin can alleviate diabetic retinal damage through its antioxidant effects. In the early stages of diabetes, enhanced retinal oxidative stress induces adaptive activation of the nuclear factor E2-related factor 2 (Nrf2) pathway. Curcumin down-regulates oxidative stress-induced Nrf2, MDA, HO-1; up-regulates SOD, T-GSH. When curcumin is combined with insulin, the effect of maintaining Nrf2 pathway homeostasis in diabetic rats is better than that of insulin alone. Transcriptomic analysis showed that curcumin, alone or in combination with insulin, inhibited the AGE-RAGE signaling pathway and extracellular matrix-receptor interaction in the diabetic retina. Therefore, in the early stages of diabetes, curcumin can be used as a potential complementary treatment option in combination with antihyperglycemic drugs for more effective treatment of diabetic complications (Xie et al., 2021). In addition, curcumin can also inhibit the damage of inflammation and oxidative stress to RPE cells by reducing inflammatory factors such as TNF-α, IL-6, and IL-1β, and reducing ROS, Akt, mTOR phosphorylation (Ran et al., 2019).

Puerarin can effectively slow down the apoptosis of retinal ganglion cells and improve the progression of type 2 DR in rats with type 2 diabetes, and its mechanism may be related to down-regulating the levels of MDA, IL-1β, TNF-α, MDA, Nrf2, p-ERK, P53. Upregulation of SOD, Bcl-2 was associated with reducing inflammation and oxidative stress in retinal tissue (Zhang and Wang, 2019).

Wogonin has a variety of biological activities and effects, such as hypoglycemia, neuroprotection, anti-tumor, etc., among which the role of hypoglycemia and prevention and treatment of diabetic complications has been paid more and more attention (Chen et al., 2022). After treatment with baicalein, the serum VEGF and CTGF levels of diabetic mouse models were increased, and the SOD activity was increased. The possible mechanism is as follows: baicalein plays an important role in regulating ROS levels, and through its antioxidant effect, it scavenges oxygen-free radicals and lipid oxidation processes and protects blood vessels and nerves (Hou et al., 2019).

Luteolin can prevent retinal cell apoptosis in rats with DR, and its mechanism may be related to NLRP/NOX4 signaling pathway, and alleviate the validation response and oxidative stress of retinal tissue by downregulating MDA, SOD, NLRP1, NOX4, TXNIP, NLRP3 (Yang et al., 2021).

Quercetin is a natural flavonoid with a wide range of pharmacological effects, including hypoglycemic effects, antioxidant, anti-inflammatory, anti-apoptotic, and immunomodulatory effects (Cheng et al., 2019). The diabetes model of male SD rats was established by intraperitoneal injection of streptozotocin, and they were randomly divided into model group, Quercetin low dose group and Quercetin high dose group. Normal rats were used as control group. Quercetin group was intraperitoneally injected with Quercetin every day, the low-dose group was 10 mg/kg, the high-dose group was 50 mg/kg. Normal control group and diabetic group were not treated for 12 weeks. The results showed that the expression of Bcl-2 protein decreased gradually in low dose and high dose Quercetin groups, while the expression of Bax and P53 protein increased significantly (Zhu et al., 2023; Wang et al., 2021). In addition, quercetin can also increase CAT, and GSH, reduce NF-kB, caspase-3, GFAP, and AQP4, and can effectively protect against diabetes-induced retinal neurodegeneration and oxidative stress (Kumar et al., 2014).

Blueberry anthocyanins can inhibit the development or progression of retinopathy, and its mechanism may be related to the down-regulation of ROS, MDA, TNF-α, and IL-1β levels; up-regulating the contents of GSH, HO-1, Nrf2, and GPx was associated with reducing inflammation and oxidative stress (Song et al., 2016).

Resveratrol is a polyphenolic plant metabolite, which is widely found in a variety of plants such as veratrol, grape, and Chinese knotweed, which can effectively activate Nrf2, thereby promoting antioxidant function and reducing oxidative stress in DR retinal tissue (Jiménez-Osorio et al., 2015). Li et al. showed that resveratrol can reduce oxidative stress and alleviate the apoptosis of retinal capillary endothelial cells by activating the AMPK/Sirt1/PGC-1α pathway and down-regulating ROS levels (Li et al., 2017). In addition, resveratrol can also reduce endothelial cell-to-mesenchymal transition and reduce NADPH to attenuate retinal damage caused by oxidative stress (Giordo et al., 2021).

Piceatannol is a derivative of resveratrol, which has been pharmacologically proven to have hypoglycemic and powerful antioxidant and free radical scavenging effects (Kotake et al., 1998). Studies have shown that piceatannol attenuates retinal ganglion cell damage in diabetic rats and is associated with the activation of the Nrf2/HO-1 signaling pathway by reducing MDA levels; The contents of SOD, Nrf2, and HO-1 were increased, and the oxidative stress of retina in DR rats was reduced (Luo et al., 2021).

Astragaloside IV is one of the active metabolites of the traditional Chinese medicine *Astragalus sinicus* L, which has strong immunostimulating, anti-inflammatory, and antioxidant effects (Fan Y. et al., 2017; Zhang R. et al., 2019). Studies have shown that astragaloside IV protects rat retinal capillary endothelial cells from high glucose-induced oxidative damage, and its mechanism may be related to the downregulation of ROS, MDA, Nox4, NADPH; It is related to the increase in the expression of antioxidant factors such as SOD, MnSOD CAT, GSH-PX, and GSH (Qiao et al., 2017). Astragaloside IV can improve early DR symptoms, significantly reduce the expression of IL-6 and TNF-α, improve the activity of SOD, and reduce the content of MDA in rats with type 1 diabetes. Its mechanism of action is related to the inhibition of inflammation, oxidative stress, and regulation of retinal VEGFA expression level (Jiang et al., 2023). Astragaloside IV can alleviate oxidative stress and inflammatory damage in the retina of type 2 diabetic rats, and its mechanism may be related to the improvement of SOD, CAT, and GSH activity or content in the retina of rats. Decrease IL-6, TNF-α, VCAM-1, HIF-1α, VEGF-A, and IL-1β to alleviate and delay the progression of DR (Li et al., 2024). In addition, astragaloside IV can activate the Nrf2-ARE pathway, increase the transcriptional activity of Nrf2, inhibit the transcriptional activity of NF-κB, and improve the oxidative stress state of the retina of diabetic rats (Yu et al., 2020).

Wogonoside is the main flavonoid active metabolite of Scutellaria baicalensis in Coptis Jiedu Soup, which has anti-inflammatory and antioxidant effects (He et al., 2014). Shao et al. constructed an *in vitro* DR model by inducing high glucose in human retinal microvascular endothelial cells, an STZ-induced DR model in SD rats. The results showed that wogonoside might alleviate the abnormal proliferation, migration, angiogenesis, cell membrane permeability, oxidative stress, and inflammatory response of hyperglycemia-induced hRMECs by up-regulating the expression of SIRT1, GSH-ST, and down-regulating the levels of ROS, NO, IL-1β, IL-6, VEGF, and HIF-1α (Shao et al., 2022). At the same time, the treatment of baicalin also significantly increased the expression level of srit1 in the retinal tissue of the DR model of SD rats.

**FIGURE 2:**
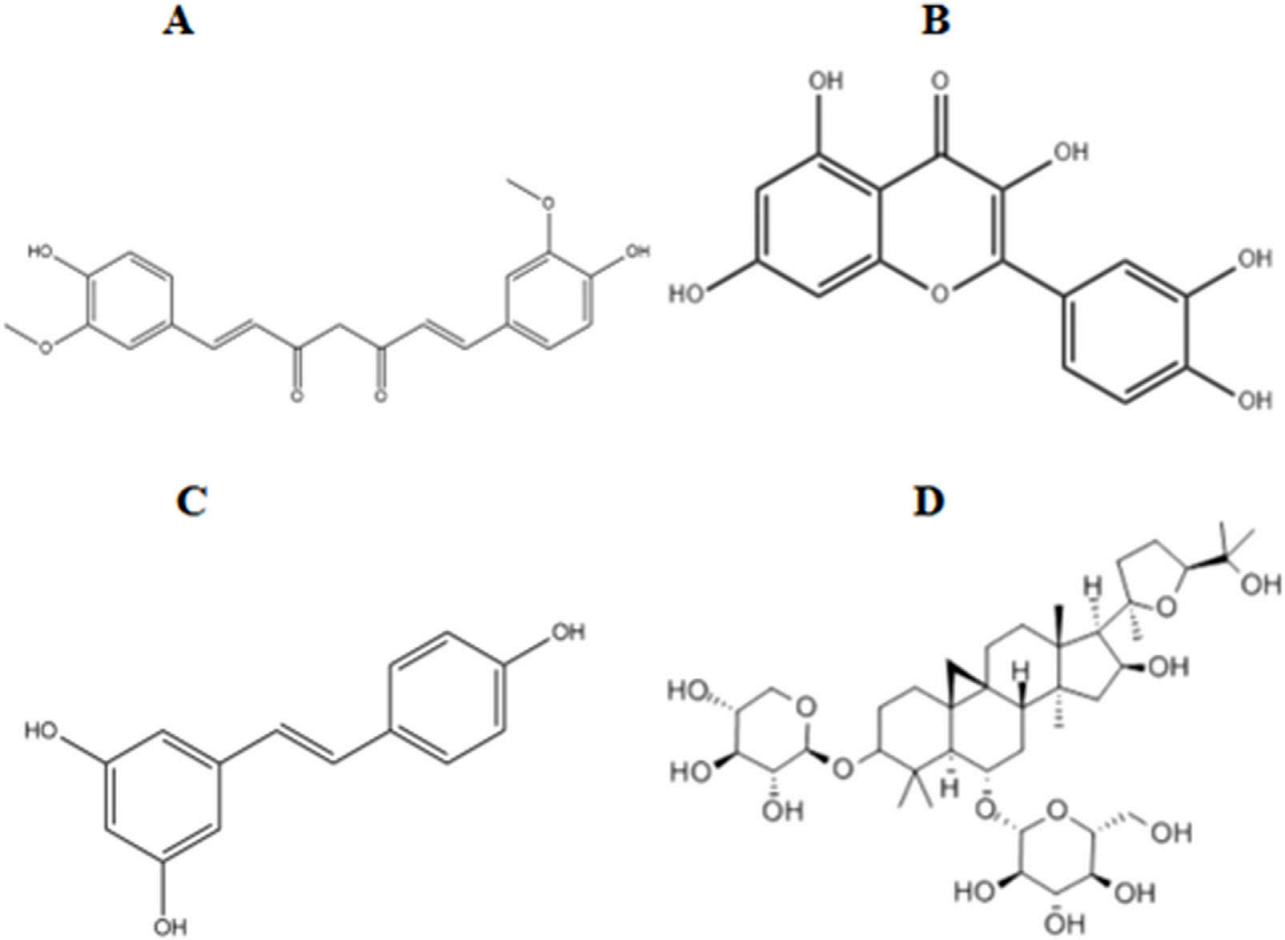
Chemical structures of the metabolites of several polyphenols. **(A)** Curcumin. **(B)** Quercetin. **(C)** Resveratrol. **(D)** Astragaloside IV.

Paeonol is a natural phenolic plant metabolites, and modern pharmacological studies have shown that paeonol has anti-inflammatory, antioxidant, and antidepressant effects (Du et al., 2024). Oxidative stress in DR is reduced by increasing the levels of GSH, SOD, and CAT and decreasing the levels of MDA (Adki and Kulkarni, 2022) (Figure 2).

### 3.2 Polysaccharides

Lycium barbarum L has the effect of nourishing the liver and kidney, benefiting the shrewd purpose, and is mainly used for the treatment of liver and kidney yin deficiency syndrome. According to the “Compendium of Materia Medica”, Lycium barbarum L has the effect of “nourishing the kidneys, moistening the lungs, and brightening the eyes”. Lycium barbarum polysaccharide (LBP) is the main active metabolite of Lycium barbarum. In recent years, pharmacological studies have confirmed that LBP has antioxidant, anti-aging, immunomodulatory, and anti-fatigue functions (Tian et al., 2019), and has obvious antagonistic effects on oxidative stress and inflammatory responses (Fang et al., 2016; Du et al., 2016; Huang, 2020). Studies have shown that LBP can increase the activity of SOD and reduce the expression of VEGF, VEGF mRNA, and MDA in rats with DR after treatment, indicating that LBP can inhibit neuronal apoptosis through antioxidant effect (Guo et al., 2015). In addition, Zhang et al. (2016) used intraperitoneal injection of 55 mg/kg streptozotocin (STZ) to induce diabetes while administering 250 mg/kg LBP by gavage for 10 weeks. The results indicated that LBP could attenuate the progression of DR by downregulating ROS, MDA, VEGF165, VEGFR1, VEGFR2, VEGFR3, Ang-1, Ang-2, Tie-1, Tie-2, TNF-α, IL-1β, ICAM-1, AOOP, and 8-OHdG; and upregulating T-AOC. This intervention suppressed retinal angiogenesis, oxidative stress, and inflammatory responses in diabetic mice (Table 2).

**TABLE 2:** Application of polysaccharides in reducing oxidative stress in the treatment of diabetic retinopathy.

Polysaccharides	Composition	Models	Administration	Mechanism	Reference
Lycium barbarum polysaccharide	Ara:Rha:Xyl:Man:Gal:GLu = 13.19:3.41:2.92:10.53:18.92:9.82	STZ-induced diabetic rats	250 mg/kg	↓: ROS, MDA, VEGF165/R1/R2/R3, Ang-1/2, Tie-1/2, TNF-α, IL-1β, ICAM-1, P-selectin, AOOP, 8-OHdG	Gao et al. (2021)
		STZ-induced diabetic rats	0.5 mL 6% LBP gastric lavage	↓: MDA; ↑:SOD	Zhang et al. (2016)
Astragalus polysaccharides	APS-Ⅰ:Rha:GalA:Glu:Gal:Ara = 0.1∶0.39∶17.2∶13.4∶1; APS-Ⅱ:Rha:GalA:Glu:Gal:Ara = 0.14∶0.14∶24.04∶9.6∶1	Human ARPE-19 and rat PRPE	12.5、20、50 μg/mL	↓: ROS, MDA, Bax, Caspase-3/9; ↑: SOD, GPx, Bcl-2	Cao et al. (2023)
Polygonatum sibiricum polysaccharides		Human ARPE-19 cells	0, 6.25,12.5, and 25 mL	↓: ROS, MDA; ↑: SOD, GPx	Cao et al. (2023)
Angelica polysaccharide	Man:Rha:GluA:GalA:Glu:Gal:Ara = 0.57:1.00:0.13:3.06:8.16:7.17:12.69	SD rat retinal ganglion cells RGC-5	100 μmol/L	↓: MDA, Akt mRNA, GSK-3β mRNA, Bax, Caspase-3/9; ↑: SOD,GSH-Px,Nrf2 mRNA	Lai et al. (2023)
		STZ-induced diabetic rats	15、30 mg/kg	↓: MDA, Caspase-1/4/5; ↑: SOD, GPx	Li et al. (2022)
		SD rat RGC-5	100、200、400 mg/L	↓: MDA, Bax; ↑: SOD, Bcl-2	Wang et al. (2023b)
Panaxnotoginseng polysaccharides		STZ-induced diabetic rats	75、150、300 mg/kg	↑: GSH, NO, VEGF, iNOS	Meng et al. (2018)

Astragalus polysaccharides are one of the main metabolites of *Astragalus sinicus* L., which has attracted more and more attention in the study of DR in recent years. Astragalus polysaccharides intervene in the progression of diabetic retinopathy through a variety of biological mechanisms, showing significant therapeutic potential. These mechanisms include inhibition of retinal pigment epithelium cell apoptosis, attenuation of oxidative stress damage to retinal ganglion cells, and inhibition of retinal neovascularization (Gao et al., 2021). Specifically, APS can reduce the levels of MDA, Bcl-2, Bax, Caspase-3, and caspase-9 in hyper glycogen-induced retinal pigment epithelial cells. At the same time, the expression of SOD was upregulated to alleviate oxidative stress and apoptosis caused by mitochondrial dysfunction in DR (Liu et al., 2019).

Polygonatum sibiricum polysaccharide (PSP) is one of the main metabolites of traditional Chinese medicine Polygonatum sibiricum Delar. ex Redoute. Studies have shown that PSP is widely involved in physiological processes such as regulating blood glucose, blood lipids, immune function, oxidative stress, etc., and has pharmacological effects such as antioxidant, hypoglycemic, hypolipidemic, immune regulation, anti-inflammatory, and antibacterial (Zhao et al., 2022). PSP alleviates high glucose-induced oxidative stress by regulating the Nrf2/HO-1 signaling pathway, reducing ROS production and MDA content, and increasing the activity of SOD and GPx. In addition, PSP significantly increased bcl-2 expression and decreased Bax expression and caspase-3 activity. Protects ARPE-19 cells from HG-induced oxidative stress, inflammation, and apoptosis (Wang W. et al., 2022).

Angelica polysaccharide is an extract of Angelica japonica A. Gray, a traditional Chinese medicine in China, which has anti-inflammatory, immunomodulatory, antioxidant, and other effects. Cao et al. (2023) used high glucose to induce RGC cell line RGC-5 to establish a model, and the results showed that Angelica sinensis polysaccharides could improve SOD activity and Bcl-2 protein expression. Reduce MDA level and Bax expression, thereby alleviating the damage of RGC oxidative stress induced by high glucose and reducing apoptosis (Wang Y. X. et al., 2023). In addition, the research group also found that Angelica sinensis polysaccharides can inhibit hyperglucose-induced apoptosis in RGC-5 cells, reduce the level of intracellular oxidative stress, and increase the expression level of intracellular antioxidant factor Nrf2 by blocking the Akt/GSK-3β pathway. The levels of MDA, Caspase-1, Caspase-4, and Caspase-5 were decreased, and the levels of SOD and GPx were increased after treatment with STZ-induced DR in Angelica sinensis (Lai et al., 2023).

**FIGURE 3:**
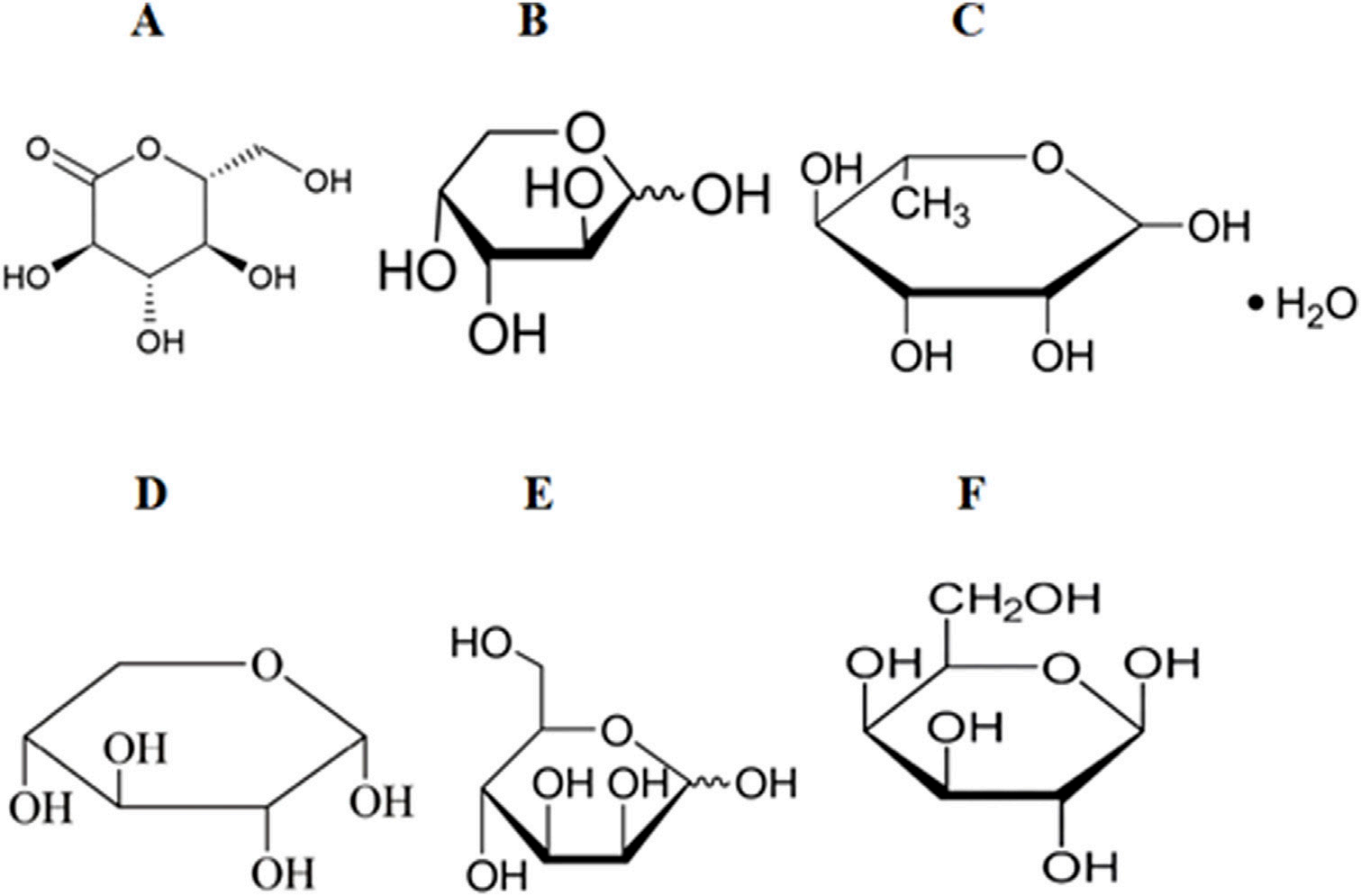
Chemical structures of the metabolites of several polysaccharides. **(A)** glucose (Glu). **(B)** L-Arabinose (Ara). **(C)** L-rhamnose monohydrate (Rha). **(D)** Xylose (Xyl). **(E)** D-Mannose (Man). **(F)** Galactose (Gal).

Panax notoginseng polysaccharide is one of the main active metabolites of Panax notoginseng (Burkill) F. H. Chen ex C. H. Chow in botanical drugs, which has attracted extensive attention in botanical drugs research in recent years. Studies have shown that Panax notoginseng polysaccharides can play a positive health role in many aspects, can reduce blood sugar levels, and can be used as an adjuvant treatment for diabetes. Panax notoginseng polysaccharide has good antioxidant activity, which can scavenge free radicals and reduce the negative effects of oxidative stress, thereby protecting the normal function and health of cells (Li et al., 2022; Wu and Wang, 2009). Studies have shown that Panax notoginseng polysaccharides can improve retinopathy caused by diabetes by increasing GSH and NO and increasing the expression of VEGF and iNOS genes (Yang et al., 2017) (Figure 3).

### 3.3 Saponins

Ginsenoside is one of the active metabolites of Panax ginseng C. A. Mey, which has anti-diabetic, antioxidant, anti-inflammatory, and anti-tumor effects (Meng et al., 2018). Wang et al. (2020) used intraperitoneal injection of STZ to establish a DR model in rats. After successful modeling, rats in the H-Rg3 and L-Rg3 groups were intraperitoneally injected once daily with 5.0 mg·kg^−1^ and 0.5 mg·kg^−1^ of Rg3, respectively, for a total of 28 injections over 28 days. The results showed that ginsenoside Rg3 could protect the retinal tissue of diabetic retinopathy rats by downregulating MDA, LDH, caspase-3, VEGF, and ICAM-1, and upregulating Bcl-2, SOD, PI3K, and p-Akt. Ginsenoside Re can also reduce the content of ROS, MDA, LDH, caspase-3, caspase-9, HIF-1α, VEGF, etc. Increase CAT, GSH-Px, and p-Akt content to ameliorate oxidative stress in retinal vascular endothelial cells in monkeys (Xie et al., 2020). Ginsenoside Rb1 can be used to target the redox state in RCEC by activating the NMNAT-NAD-PARP-SIRT axis under high glucose conditions, which has an advantage during high glucose-induced cell damage (Fan et al., 2019). Ginsenoside Rd promotes AMPK activation and SIRT1 expression, reversing high glucose-induced NOX2 activation, oxidative stress, mitochondrial dysfunction, and endothelial cell apoptosis in an AMPK/SIRT1-dependent manner. Rd restores hyperglycemia-induced endothelial cell loss and other retinal damage *in vivo* by activating AMPK and SIRT1, which significantly modulates oxidative stress and apoptosis to ameliorate diabetes-driven vascular injury (Tang et al., 2022) (Table 3).

**TABLE 3:** Application of saponins in the treatment of diabetic retinopathy by reducing oxidative stress.

Saponins	Chemical formula	Models	Administration	Mechanism	Reference
Ginsenoside Rg3	C42H72O13	STZ-induced diabetic rats	0.5、5.0 mg/kg	↓: MDA, LDH, Caspase-3, VEGF, ICAM-1; ↑: Bcl-2, SOD, PI3K, p-Akt	Xie et al. (2020)
Ginsenoside Re	C48H82O18	The monkey RF/6A cells	3 μM	↓: ROS, MDA, LDH, caspase-3/9, VEGF, HIF-1α; ↑: CAT, GSH-Px, P-Akt	Fan et al. (2019)
Ginsenoside Rb1	C54H92O23	RCECs	5、10 、20 μM	↓: ROS, NOX, PARP; ↑: CAT, SOD, SIRT1/3	Tang et al. (2022)
Ginsenoside Rd	C48H82O18	STZ-induced diabetic rats	100 μM	↓: ROS, NOX2, 8-OHdG; ↑: AMPK, SIRT1, NAD/NADH	Fan et al. (2017b)
Notoginsenoside R1	C47H80O17	RCECs	5、10 、20 μM	↓: ROS, 3-NT, Nox, PARP, ICAM-1, Bad; ↑: CAT, NADPH, GSH	Zhou et al. (2020)
		rMC-1 cells; db/db mice	5、10、20 、40 μM; 30 mg/kg	↓: ROS, 4-HNE, protein carbonyl, 8-OHdG, VEGF; ↑: PEDF	Fan et al. (2016)
		SD rats	50、100、200 μg/mL	↓: ROS, MDA, Nox4; ↑: SOD, MnSOD, CAT, GSH-PX activities; GSH	Fang et al. (2019)

**FIGURE 4:**
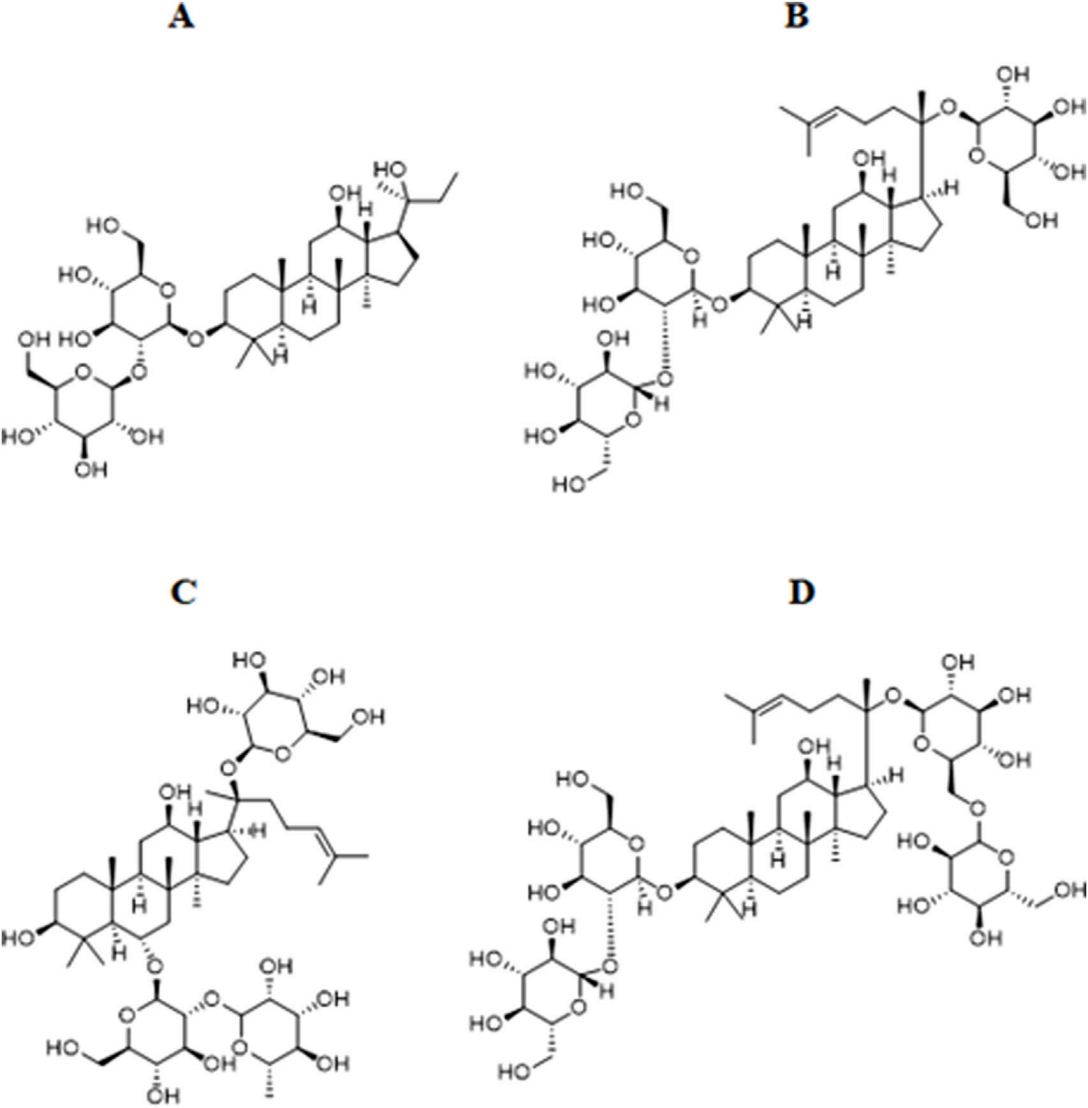
Chemical structural formula of the representative saponin metabolites of Panax ginseng. **(A)** Ginsenoside Rg3. **(B)** Ginsenoside Rd. **(C)** Ginsenoside Re. **(D)** Ginsenoside Rb1.

Notoginsenoside R1 is derived from the dried roots and rhizomes of Panax notoginseng (Burkill) F. H. Chen ex C. H. Chow and has antioxidant, anti-cancer, and cardiovascular disease effects. Notoginsenoside R1 attenuates hyperglucose-induced damage to retinal capillary endothelial cells (RCECs), reduces oxidative stress, and inhibits redox state changes in RCECs (Fan C. et al., 2017). Db/db mouse experiments showed that Notoginsenoside R1 could inhibit the apoptosis of rat retinal Müller cells under high glucose, significantly inhibit the expression of VEGF, increase the expression of PEDF, and inhibit oxidative stress and inflammation (Zhou et al., 2020). Notoginsenoside R1 can also increase the activities of the retina, total SOD, MnSOD, CAT, and GSH-PX in SD rats, directly reducing the damage of oxidative stress to the retina (Fan et al., 2016) (Figure 4).

### 3.4 Alkaloids

Evodiamine (EVO) is an alkaloid extracted from the unripe dried fruits of Evodiamine in traditional Chinese medicine and has a variety of pharmacological activities, including hypolipidemic, anti-inflammatory, anti-infective, and antitumor effects (Fang et al., 2019; Shin et al., 2007). In addition, EVO also has a significant effect on diabetes, it can reduce blood sugar levels in diabetic rats, and improve oxidative stress and inflammatory responses (Yu et al., 2022). EVO can reduce the content of MDA, TNF-α, IL-6, Bax, and P53 in retinal tissues of STZ-induced DR models, and the reduction of these biomarkers suggests that evodiamine effectively alleviates oxidative stress and inflammation in retinal tissues. At the same time, EVO also further exerts its protective efficacy by up-regulating the levels of SOD, cAMP, p-PKA/PKA, and can effectively protect the retina of DR model rats from oxidative stress and inflammation (Liu et al., 2024) (Table 4).

**TABLE 4:** Application of alkaloids in reducing oxidative stress in the treatment of diabetic retinopathy.

Alkaloids	Chemical formula	Models	Administration	Mechanism	Reference
Evodiamine	C19H17N3O	STZ-induced diabetic rats	10、20、40 mg/kg	↓: MDA, TNF-α, IL-6, Bax, P53; ↑: SOD, cAMP, p-PKA/PKA	Pang et al. (2015)
Berberine	C20H18NO4+	human Müller cells	5 μM	↓: ROS, Nox4, ICAM-1, IL-6/8, TNF-α, GFAP; ↑: Gpx-1, iNOS, Nrf2, AMPK	Zhang and Shen (2020)
Matrine	C15H24N2O	STZ-induced diabetic rats	30、60、90 mg/kg/d	↓: MDA, CRP, TNF-α, IL-1β, IL-6; ↑: SOD1	Wang et al. (2023b)
		SD rat RPECs	25、50、100 mg/L	↓: TNF-α, IL-1α, IL-6; ↑: SOD-1、Nrf2、γ-GCS、NQO1	Wang et al. (2023b)

Berberine is a natural plant metabolite proposed from the Coptis chinensis Franch. Coptis chinensis. Pharmacological studies have shown that berberine has anti-inflammatory, antioxidant, and hypoglycemic effects. Berberine is a natural plant metabolite proposed from the traditional Chinese medicine Coptis chinensis. Pharmacological studies have shown that berberine has anti-inflammatory, antioxidant, and hypoglycemic effects (Pang et al., 2015), which makes it a potential treatment option for a variety of health problems. In particular, berberine has shown positive effects in ophthalmic studies. One study found that berberine was effective in reducing ROS, Nox4, ICAM-1, IL-6, IL-8, TNF-α, GFAP in highly oxidized and glycated (HOG-) low-density lipoprotein (HOG-LDL) cultured Müller cells; Upregulation of Gpx-1, iNOS, Nrf2, and AMPK inhibits modified LDL-induced Müller cell damage by activating the AMPK pathway, and plays a role in the prevention or treatment of DR (Fu et al., 2016).

**FIGURE 5:**
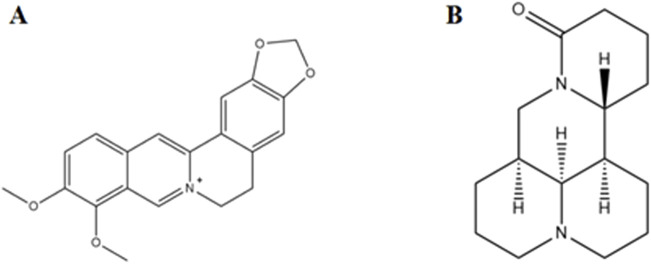
Chemical structural formula of the alkaloids. **(A)** Berberine. **(B)** Matrine.

Matrine is one of the metabolites of Sophora flavescens Aiton, and pharmacological studies have shown that matrine and oxidized matrine have a wide range of biological activities, including antibacterial, antiviral, antioxidant, anti-inflammatory, and immunomodulatory (Zhang and Shen, 2020). By regulating the expression of VEGF and ANG1, marine increased the level of SOD1 and reduced the content of MDA, CRP, TNF-α, IL-1β, and IL-6, thereby improving retinal damage and inhibiting the proliferation of retinal microvascular endothelial cells in DR rats (Zhang, 2019). In addition, at concentrations of 25, 50, and 100 mg/L, oxymatrine could resist the high glucose environment, inhibit the decreased activity of rat retinal pigment epithelial cells cultured *in vitro*, reduce the cell damage caused by high glucose, and upregulate the expressions of Nrf2, SOD-1, γ-GCS and NQO1, and downregulate the expressions of TNF-α, IL-1α and IL-6 (Zhang, 2015). Both marine and oxymatrine have hypoglycemic effects (Zhang, 2015; Yan, 2010; Zhang et al., 2019c), suggesting that they help prevent and treat diabetic retinopathy through their basic pharmacological effects of antioxidant, anti-inflammatory, and hypoglycemic effects (Figure 5).

### 3.5 Characteristics of plant metabolites to improve oxidative stress of DR

**FIGURE 6:**
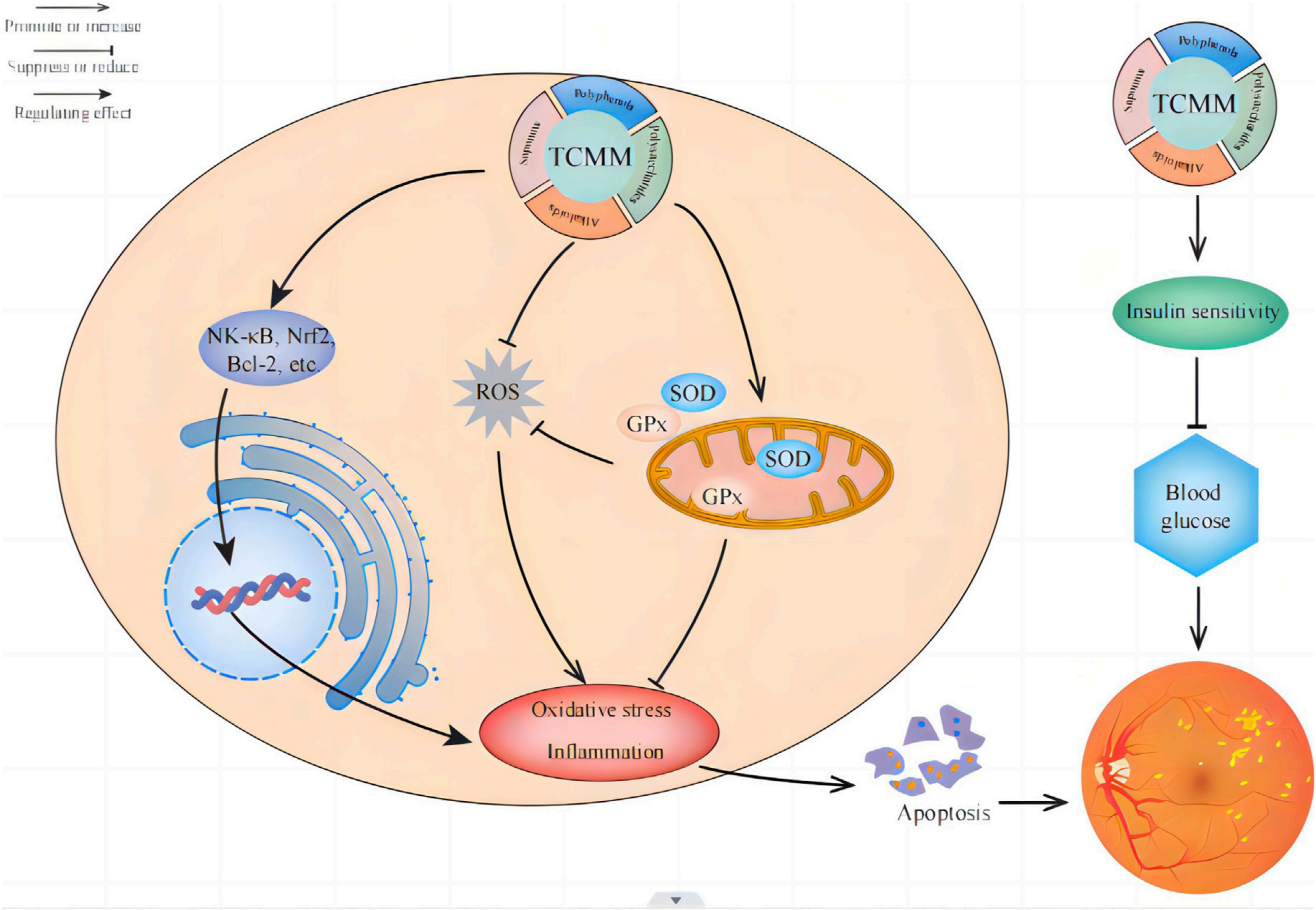
Application of plant metabolites in reducing oxidative stress in the treatment of diabetic retinopathy. There are four main types of plant metabolites (polyphenols, polysaccharides, saponins, and alkaloids) to improve diabetic retinopathy, which can alleviate the progression of diabetic retinopathy by enhancing antioxidant capacity, promoting the synthesis of endogenous antioxidants, improving blood sugar control, and inhibiting retinal cell apoptosis.

In summary, plant metabolites have shown the following characteristics in improving diabetic retinal oxidative stress: (1) Multi-target effect: plant metabolites can act on multiple biological targets at the same time, and reduce the damage of oxidative stress to the retina by regulating related signaling pathways, such as NF-κB, Nrf2, Bcl-2, etc. (2) Strong antioxidant capacity: Many plant metabolites, such as polyphenols, polysaccharides, flavonoids, etc., have significant antioxidant properties, which can effectively scavenge free radicals and reduce oxidative damage to retinal cells. (3) Promote the synthesis of endogenous antioxidants: Some plant metabolites can promote the synthesis of antioxidant enzymes in the body, such as SOD and glutathione peroxidase, thereby enhancing the body’s natural antioxidant capacity. (4) Inhibition of inflammatory response: Plant metabolites often have anti-inflammatory effects, which can inhibit the inflammatory response in the retina, reduce the expression of inflammatory factors, and indirectly reduce the damage of oxidative stress to the retina. (5) Improve blood sugar control: Some plant metabolites such as astragalus polysaccharides and ginsenosides can improve insulin sensitivity and reduce blood sugar levels, thereby reducing chronic complications caused by diabetes, including retinopathy. (6) Protection of retinal cells: Plant metabolites protect retinal nerve cells by inhibiting retinal nerve cell apoptosis promoting cell survival, and directly alleviating the progression of diabetic retinopathy (Wang Y. X. et al., 2023). These characteristics make plant metabolites have significant potential and advantages in the prevention and treatment of diabetic retinal oxidative stress (Figure 6).

## 4 Conclusions and prospects

### 4.1 Research status of plant metabolites

In recent years, the significant antioxidant capacity of many plant metabolites has been confirmed, and more and more studies have revealed that plant metabolites can play a protective role in diabetic retinopathy by regulating multiple biological targets and related signaling pathways to promote the expression of antioxidant enzymes. In terms of animal studies, several animal experiments have shown that plant metabolites can significantly improve retinopathy and reduce retinal damage indicators in diabetic mice and rats. In terms of clinical studies, although relatively few, there have been some small-scale clinical trials that have shown that plant metabolites have a certain improvement effect on retinopathy in diabetic patients.

### 4.2 Study limitations

At present, there are still the following limitations: (1) Small sample size: The sample size of many studies is small, and the statistical significance and clinical applicability of the results need to be further verified. (2) Lack of long-term research: At present, most of the studies are on the short-term use of plant metabolites, such as 1 month to 1 year, and there are still few studies on the safety and effect of long-term use. (3) Standardization problem: There is no unified standard for the extraction, purification, and quality control of plant metabolites, which affects the reproducibility and reliability of research results. (4) Insufficient mechanism exploration: Although some mechanisms have been explored, the interaction mechanism between various plant metabolites still needs to be further explored, especially in the context of specific complex diabetic retinopathy. (5) Incomplete safety evaluation: Although plant metabolites are generally considered to be relatively safe, there may be potential adverse reactions when used in the treatment of DR For a long time or at high doses. For example, tetramethy lpyrazine, a botanical drug that has the effect of promoting blood circulation and removing blood stasis, may increase the risk of eye bleeding while improving eye microcirculation. At present, the evaluation in this regard is insufficient.

### 4.3 Future directions

In view of the above limitations, the following aspects can be used in the future: (1) Carry out multi-center large-scale clinical trials: carry out multi-center and large-sample clinical studies, and do a good job in statistical analysis and clinical efficacy observation. (2) Conducting long-term prospective studies over several years to decades, to verify the efficacy and safety of plant metabolites for diabetic retinopathy. (3) Standardization and formulation research: Establish a standardized extraction and quality control system for plant metabolites, and develop more effective preparations to improve the clinical application effect. (4) In-depth research on mechanism: Strengthen the basic research on the mechanism of action of plant metabolites, and explore its interactive biological effects and multiple signaling pathways in diabetic retinopathy. Combined with modern technology, such as genomics, proteomics, etc., the mechanism of action of plant metabolites was deeply explored, and the interaction mechanism between different plant metabolites was sought. (5) The toxicology of plant metabolites, including acute toxicity, long-term toxicity and genetic toxicity, will be studied systematically. At the same time, attention should be paid to the safety of drugs in special groups (such as the elderly, pregnant women, etc.).

Through these efforts, it is expected that the potential of plant metabolites in improving diabetic retinopathy can be better understood and applied.

## Abbreviations

DR, Diabetic Retinopathy; ROS, Reactive Oxygen Species; OS, Oxygen species; GPx, Glutathione peroxidase; MDA, Malondialdehyde; SOD, Superoxide dismutase; CAT, Catalase; AGE, Advanced glycation end products; HO-1, Heme oxygenase-1; ICAM, Intercellular adhesion molecule; HIF-1α, Hypoxia inducible factor-1α; Nrf2, Nuclear factor E2-related factor 2; LBP, Lycium barbarum polysaccharide; RCECs, Retinal capillary endothelial cells; EVO, Evodiamine.
